# Are Front-of-Package Warning Labels More Effective at Communicating Nutrition Information than Traffic-Light Labels? A Randomized Controlled Experiment in a Brazilian Sample

**DOI:** 10.3390/nu10060688

**Published:** 2018-05-28

**Authors:** Neha Khandpur, Priscila de Morais Sato, Laís Amaral Mais, Ana Paula Bortoletto Martins, Carla Galvão Spinillo, Mariana Tarricone Garcia, Carlos Felipe Urquizar Rojas, Patrícia Constante Jaime

**Affiliations:** 1 Center for Epidemiological Studies in Health and Nutrition (NUPENS), Faculty of Public Health, University of São Paulo, Av. Dr. Arnaldo, 715-Cerqueira César, São Paulo 01246-904, Brazil; pri.sato@gmail.com (P.d.M.S.); constant@usp.br (P.C.J.); 2 Brazilian Institute for Consumer’s Defense (Idec), R. Desembargador Guimarães, 21-Água Branca, São Paulo 05002-000, Brazil; lais.amaral@idec.org.br (L.A.M.); anapaula@idec.org.br (A.P.B.M.); marianatarricone@gmail.com (M.T.G.); 3 Research Group of Digital and Information Design, Department of Design, Federal University of Paraná, Rua General Cameiro, 460, Curitiba 80060-050, Brazil; cgspin@gmail.com (C.G.S.); chilenus@gmail.com (C.F.U.R.)

**Keywords:** warning labels, traffic-light labels, randomized controlled experiment, Brazil, front-of-package labels, health promotion

## Abstract

Background: Brazil is currently debating the implementation of front-of-package labels. This study tested if Warning labels (WLs) improved consumer understanding, perceptions, and purchase intentions compared to Traffic-Light labels (TLLs) in 1607 Brazilian adults. Methods: In this online, randomized controlled experiment participants saw images of 10 products and answered questions twice—once in a no-label, control condition and then again in a randomly assigned label condition. The relative differences in responses between WLs and TLLs between control and label conditions were estimated using one-way ANOVAs or Chi-square tests. Results: Presenting WLs on products compared to TLLs helped participants: (i) improve their understanding of excess nutrient content (27.0% versus 8.2%, *p* < 0.001); (ii) improve their ability to identify the healthier product (24.6% versus 3.3%, *p* < 0.001); (iii) decrease perceptions of product healthfulness; and (iv) correctly identify healthier products (14.0% versus 6.9%, *p* < 0.001), relative to the control condition. With WLs, there was also an increase in the percentage of people: (v) expressing an intention to purchase the relatively healthier option (16.1% versus 9.8%, *p* < 0.001); and (vi) choosing not to buy either product (13.0% versus 2.9%, *p* < 0.001), relative to the control condition. The participants in the WL condition had significantly more favorable opinions of the labels compared to those in the TLL group. Conclusions: WLs would be more effective, compared to the TLL, at improving consumer food choices.

## 1. Background

Front-of-package (FOP) labels provide consumers with easy-to-understand, concise information about the nutrient profile of a food product [1]. Health agencies endorse FOP labels as a crucial policy measure for informing consumer food purchasing practices and encouraging healthier food choices. These food choices reduce the consumption of products with excessive sugars, sodium, and saturated fat [2]—nutrients implicated in the causation of obesity and cardiovascular disease [3]. Indeed, providing nutrition information at points of purchase through labels is likely to be one of the few cost-effective strategies for supporting a healthy dietary pattern that may protect against future non-communicable diseases [4,5]. 

FOP labels that are currently in use vary in their degree of regulation, ranging from voluntary recommendations, as seen in the UK, Australia, and Europe, to mandatory policies implemented in Chile, Ecuador, Mexico, Peru, Israel, and Thailand [6]. FOP labels also vary in their format and design. Some integrative systems use symbols or logos to indicate relatively healthy products, while nutrient-specific labels display the amount of key nutrients [7]. The UK Traffic-Light label (TLL) is an example of the latter system. It displays calories, sugar, fat, saturated fat, and salt per portion of product, and their equivalent percentage contribution to an adult’s daily needs [8]. Color codes indicate if the nutrient is present in high (red), medium (amber), or low (green) amounts. Warning labels (WLs) are a more recent format of nutrient-specific FOP labels that are only displayed when key nutrients exceed the recommended levels. The WL model implemented in Chile combines a simple text and an easily recognizable octagonal ‘stop’ symbol in black-and-white, displaying a separate WL for every nutrient that is in excess [9].

Brazil, as the first country to make specific commitments towards the United Nation’s Decade of Action on Nutrition that include implementing FOP labels [10], is currently debating the most appropriate FOP label for its citizens. The interpretative formats of the TLL and the WL are amongst the options under consideration [11]. While there is some evidence that demonstrates consumer support for the adoption of a FOP labeling policy [12], there is less clarity on the most effective format for the Brazilian population.

In their conceptual models, Grunert and colleagues highlight label understanding as a key determinant of label use and effectiveness [13,14]. This in turn influences consumer ability to distinguish less healthful products from more healthful ones and, along with label appeal, affects purchase decisions. Experimental studies have generally supported TLLs’ effectiveness in improving consumer understanding compared to less interpretive formats [15,16,17,18,19]. Consumers also demonstrate an improvement in perceptions of product healthfulness and food selection [20,21,22]. However, real-world evidence provides less support for the TLL [23]. Recent evidence comparing the TLL to more interpretative FOP labels like the WL is also less in favor of the TLL; WLs seem to have a stronger effect than TLLs in discouraging children’s choice of a snack and a juice in a conjoint experiment study [24]. However, results are mixed. Among adults, the WLs lowered the perceptions of healthfulness of a product but were no different to the TLLs in improving consumer ability to identify a healthier product [25]. The TLLs were equivalent to the WLs in improving the average nutritional composition of the shopping basket in a simulated shopping experiment [26]. The one study that was conducted within an adult Brazilian sample also found equal perceptions of product healthfulness for TLLs and WLs [27].

While there is a growing body of evidence that compares TLLs to WLs across different outcomes, evidence from the Brazilian population remains sparse. In an attempt to provide empirical evidence to inform the political and academic discourse on FOP labelling, this study aimed to evaluate the effectiveness of WLs compared to TLLs, in improving consumer understanding, perceptions, and purchase intentions, in a Brazilian sample.

Specific study objectives were to:

1. Assess if consumers were better able to determine nutrient content and product healthfulness in the presence of FOP labels;
2. Determine if the presence of FOP labels influenced purchase intentions;
3. Compare WLs and TLLs to ascertain which label was:
  - Better at indicating the presence of a nutrient above the recommended levels;
  - Better at decreasing the perceptions about the overall healthfulness of the (unhealthy) product;
  - More effective at shifting purchase intentions;
  - More positively rated by consumers.

## 2. Methods

### 2.1. Study Design

A randomized controlled experiment was used for this study. In this design, all participants were exposed to a control (no-label) condition where they saw images of products and answered questions (T1). The participants were then randomly allocated to one of two intervention conditions where they saw the same product images, this time with a label (the intervention), and responded to the identical set of questions as in T1. This design used the participants as their own control, attributing any difference in their responses to the intervention. All participants consented to participate in this study. The study received approval from the ethics committee of the University of São Paulo (68795417.6.0000.5421).

### 2.2. Study Sample and Recruitment

A research firm was contracted to recruit participants from online panels who were broadly representative of the Brazilian population with regard to their age, education levels, sex, socio-economic status (SES), and geographic region. All adults responsible for grocery purchases (sole or shared responsibility), who had no links to the food industry, who did not work in food and nutrition, and who had not previously worked with market research, were eligible to participate. The participants were blinded to the study aims. The study survey was administered through a survey platform in October 2017. A total of 3353 people initiated the survey. Of these, 1607 participants completed the survey. The remainder did not meet the eligibility criteria (29.2%), entered an incorrect response to the validation question (1.2%), did not complete the survey (12.7%), or were filtered out because the demographic quota they represented was already full (8.8%). The respondents who completed the survey did not receive any monetary compensation but entered a scoring program where they earned points exchangeable for products. All study procedures were approved by the ethics committee at the University of São Paulo and were carried out in Portuguese.

### 2.3. Study Conditions

After obtaining consent, the participants saw images of food products and responded to questions at two points in time—once while viewing images without labels (T1) and then again while viewing images with labels (T2). At T2, the participants were randomly assigned to see images of products with one of two label formats—the TLL or the WL. In this experimental study, the design, position on the product, and the nutrients displayed by the TLL were modelled on the proposal of the Brazilian Consortium of Food Industries [28], while the nutrient criteria used was informed by the UK Food Standards Agency (FSA) [29]. The design, position, nutrients, and the nutrient criteria for the WLs were based on the proposal of the Brazilian Institute for Consumer Defence [30]. The Nutrient Profile Model of the Pan-American Health Organization (PAHO) was used to determine nutrient criteria [31]. Both labels were approximately 20% of the size of the package.

**Figure 1:**
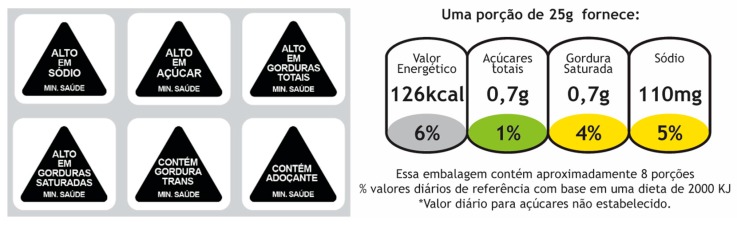
Label conditions.

**Figure 2:**
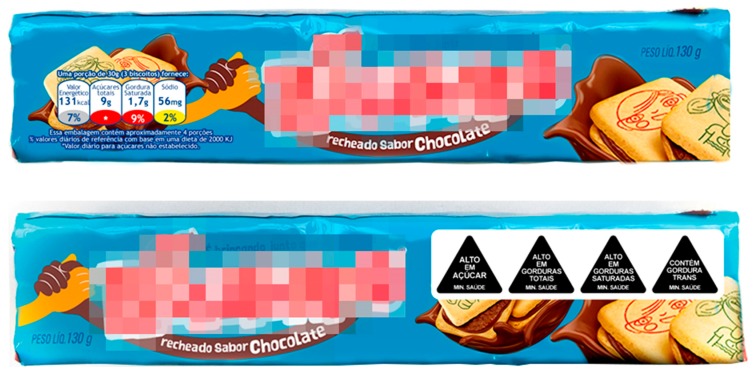
Example images of products displaying the traffic-light label and the warning labels.

(1) Traffic light labels: These displayed nutrient content by weight as well as percentages of Reference Intake (RI) per portion of the product, for total sugars (in g), saturated fat (in g), sodium (in mg), and calories (as kcals) (Figure 1). The percentage RI for calories was always represented against a grey background; however, green, amber, or red colors were used to depict low, medium, or high content for total sugars, saturated fat, and sodium, in keeping with the specifications of the FSA [29]. The actual nutrient profiles of the products were used to determine nutrient content and the combination of the three colors. TLLs appeared on all products. They were positioned at the bottom left corner of the front panel (Figure 2).
(2) Warning labels: Triangular, black-and-white WLs were used on products to indicate excess free sugars, saturated fat, total fat, or sodium, with the phrase ‘High in’ (‘Alto em’). WLs were also used to indicate the presence of *trans* fat or sweeteners, with the words ‘Contains’ (Figure 1). This WL was developed by researchers from the Department of Design at the Federal University of Paraná, Brazil, and performed better than the Chilean design in a prior study (manuscript under preparation). The actual nutrient profile of the product was used to determine which nutrients were in excess. The products carried a separate WL for every nutrient, which meant that the number of WLs differed by product. All WLs were displayed on the top right corner of the front panel (Figure 2).

### 2.4. Study Procedures

The entire study was conducted online with no contact between researchers and participants. The survey was designed to simulate decisions and tasks performed during a regular visit to the grocery store. The first part of the survey represented the control condition in which all participants saw images of the front panel of the products without labels (T1). The products selected were commonly consumed in Brazil [32] or were frequently misunderstood to be healthy, on the basis of data from the focus groups collected prior to this study (manuscript under preparation). Images of a savory snack, biscuits with chocolate-flavored filling, and flavored lemonade, created in Adobe Photoshop CC 2017, were shown one at a time. For each product, the participants were asked three questions. The participants then saw images of multiple products from different brands but from the same product category—two brands of savory biscuits, two brands of instant soups, and three brands of breakfast cereals. They responded to three questions for every combination of products (Table 1).

In the second part of the survey, the participants were randomized to a label condition (T2). To increase their familiarity with the label they responded to a series of questions (7–11, Table 1) while viewing an image of the label (Figure 1). Following this section, the participants were asked to respond to questions 1–6, in the same sequence (Table 1). All images in T2 were of the same products as T1 but displayed either a WL or a TLL. In the final section, opinions on the labels (questions 12–17) and information on participants’ chronic disease history, height, and weight were recorded.

### 2.5. Study Outcomes

#### 2.5.1. Understanding of Nutrient Content

The *Nutrient content score* (*single product*) measured the participants’ ability to identify excess nutrient content in a single product. All correctly identified nutrients in question 2 were given a score of 1, combined and converted into a mean percentage (0–100). An equivalent, *Nutrient content score* (*comparison task*)*,* was created to assess the participants’ ability to identify products with excess nutrients in a comparison task where there was more than one product to choose from. All correctly identified responses to question 5 were given a score of 1 and converted into a percentage (0–100). Only the nutrients that were identified as being in excess by both WL and TLL systems for the same products were used in the creation of these scores (i.e., displaying a triangle in the WL condition and a red cell on the TLL). This ensured that the influence of differences in nutrient criteria between labels was minimized.

#### 2.5.2. Label Understanding

Subjective label understanding was estimated from the average responses to questions 7 and 8 (Table 1).

#### 2.5.3. Product Healthfulness

For single products, the mean response to question 3 was combined and averaged across all three single products to create a single, subjective *Perceived product healthfulness* indicator. In the product comparison task, the Nutrient Profiling Model proposed by Rayner et al. [33] was used to determine the objectively healthy product of the comparison (question 6). The correct responses were given a score of 1, combined, and converted into a mean percentage to create an objective *Product healthfulness score* (0–100).

#### 2.5.4. Purchase Intentions

For single products, responses to question 1 were averaged across all three single products to create a summary response, *Purchase intentions* (*single product*). The indicator *Purchase intentions* (*comparison task*) was created by averaging the responses to question 4 across all product combinations. The mean responses to question 9 were analyzed separately.

#### 2.5.5. Label Opinions

Participant general opinion on the labels was estimated by averaging the responses from questions 10–17. Questions 14 and 17 were reverse-coded.

### 2.6. Statistical Analysis

All aggregate differences in continuous mean responses between control and label conditions were estimated using *t*-tests and one-way ANOVAs. Relative differences between WLs and TLLs between T1 and T2 were also estimated. Chi-square tests were used for categorical variables. Differences between label conditions by product type and stratification by sex, age group, education, SES, and geographical regions were also explored for any differences or deviations from the aggregate pattern. All analyses were conducted in Stata v.14 (StataCorp LLC, College Station, TX, USA).

## 3. Results

The mean age of the sample of 1607 adults was 39.2 years (±12.9). Women comprised 52.4% of the sample, 82.0% had completed primary or secondary level education, and 62.7% were from the low- or middle-SES. Both label conditions were balanced on all demographic variables (Table 2). Current dieting behavior was the only indicator that significantly differed between label conditions. Controlling for this indicator in all subsequent analysis did not change the direction or the magnitude of the results (data not presented). Additionally, stratification by sex, age group, level of education, social class, and geographic region also did not change the direction of the responses (data not presented). There were no significant differences between label conditions for the time taken to complete the survey (mean 61 min ± 4.4).

### 3.1. Do Labels Help Improve the Understanding of Nutrient Content? Which of the Labels is More Effective?

The presence of a label clearly improved the participants’ understanding of excess nutrient content in single products and increased their ability to identify products with excess nutrients. *Nutrient content scores* increased by 17.6% points on average for single products and by 14.0% for the product comparison task, compared to the control condition (Table 3).

**Figure 3:**
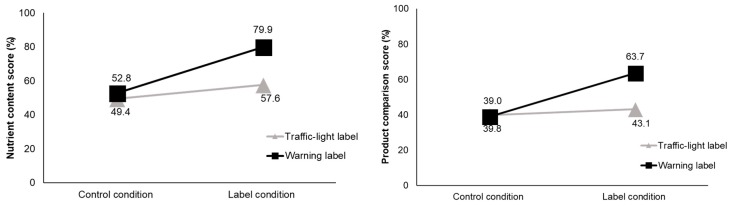
Nutrient content score (left—single product, right—comparison task); *p* < 0.001 for differences between label conditions at T2.

The extent of improvement varied between TLL and WL. While there were no differences in scores between labels at T1 as expected, there was a clear difference at T2 (Figure 3). For single products, the participants in the WL condition scored 27.0% points higher than at T1, while those in the TLL improved by 8.2% points (*p* < 0.001). Similarly, in the product comparison task, the participants in the WL condition scored 24.6% points higher than at T1, while those in the TLL improved their ability to identify products with excess nutrients by 3.3% points (*p* < 0.001, Table 4).

For subjective label understanding, the participants in the WL group chose significantly lower response options on scales of both frequency and quantity of consumption than the participants in the TLL group, indicating less frequent consumption and smaller quantities of consumption (*p* < 0.001, Table 4).

### 3.2. Do Labels Influence the Understanding of Product Healthfulness? Which of the Labels is More Effective?

The perceived product healthfulness for single products was significantly lower across the entire sample in the label condition compared to the control condition (Table 3). Again, while there were no differences between label conditions at T1, at T2 the participants in the WL condition had significantly greater decreases in perceptions of healthfulness of products compared to participants in the TLL condition.

**Figure 4:**
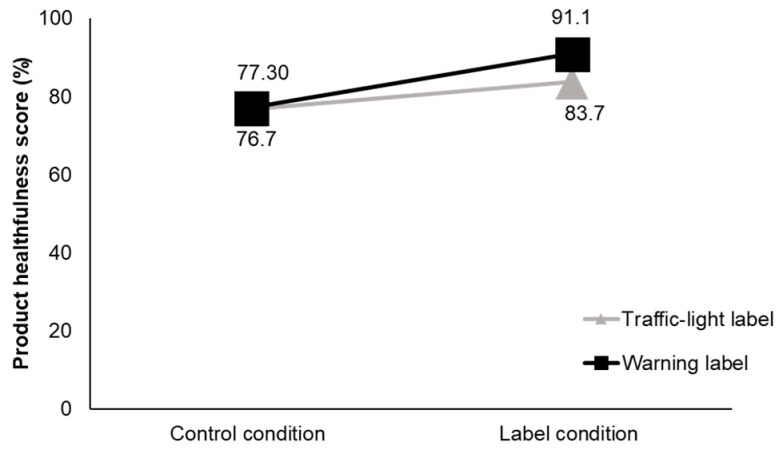
Product healthfulness score, measured in a product comparison task; *p* < 0.001 for differences between label conditions at T2.

In the product comparison task, correct responses on the product healthfulness scores were significantly higher at T2 compared to T1 (Figure 4). There were no differences between labels in correctly identifying the healthier product at T1 as anticipated. At T2, the scores of the participants in the TLL group increased by 6.9% points while those of the participants in the WL group increased by 13.7% points (*p* < 0.001). A disaggregation of results by product showed that, for soups, the percentage of participants in the TLL group who correctly identified the product with the excess nutrient decreased at T2 compared to T1. Without a label, 60.9% of participants in the TLL condition and 67.8% in the WL condition correctly identified the soup with excess sodium (*p* = 0.379). In the presence of labels, only 41.4% correct responses were reported in the TLL condition, while in the WL condition 76.7% of the participants chose correctly. Possible interpretations of this finding are discussed in Section 4.

### 3.3. Which of the Labels Are More Effective at Influencing Purchase Intentions?

When asked how the presence of the label would affect the decision to purchase a frequently bought product, the mean scores of the participants in the WL group indicated that they would be significantly less likely to continue buying the product compared to those who saw the TLL (Table 4).

The *Purchase intentions, single products* measure demonstrated a similar response pattern. There was a significant decrease in purchase intentions of the overall sample in the label condition compared to the control. While there were no differences between label conditions in intentions to purchase at T1, at T2 the participants in the WL condition had a lower mean response than those in the TL condition (*p* < 0.001). This decrease between T1 and T2 was significantly greater for the WL.

**Figure 5:**
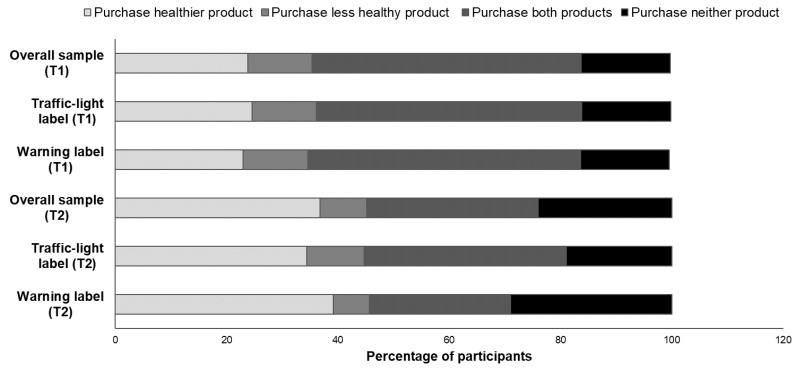
Intention to purchase, measured in a product comparison task; *p* < 0.001 for differences between label conditions at T2.

There were no differences between label conditions on the *Purchase intentions, comparison task* measure when the participants were asked about their intentions to purchase products at T1 (*p* = 0.948). Upon viewing product images with labels, two different shifts in participant choices were observed: (i) towards purchasing the healthier option, and (ii) abandoning both product options. There was a significant increase in the percentage of people choosing to purchase the relatively healthier option. In total, 34.4% of participants in the TLL condition chose the healthier option, corresponding to an increase of 9.8%, while 39.1% of participants in the WL group chose the healthier option (an increase of 16.1%). There was also a significant increase in the percentage of people who chose not to buy either product. A total of 18.9% of participants in the TL condition said they would not buy either product (an increase of 2.9% from the control condition), while 28.9% of participants in the WL condition chose this option, corresponding to an increase of 13.0% (Figure 5).

### 3.4. Which of the Labels had a More Favourable Overall Opinion?

Compared to the TLL group, participants in the WL condition had a significantly more favorable overall opinion with regards to the visibility, attention, credibility, usefulness, and ease of use of these labels (*p* < 0.001).

## 4. Discussion

The present study aimed to provide evidence on the benefits of including FOP labels on food products and to determine if the TLL or the WL was a better contender for FOP labelling in Brazil. The aggregate results demonstrate that having FOP labels was more effective than not having any labels. Between the two types of labels, WLs were consistently shown to be superior to the TLLs on all objective and subjective study outcomes.

### 4.1. Mandatory, Standardized, Front-Of-Package Labels Are Effective

In this study, participant performance on all outcomes improved in the presence of FOP labels. These results find support in the literature where generally consistent results have demonstrated that FOP labels are more effective at helping consumers identify healthier choices [20,22]. Most of the studies on FOP labels, including this one, have been lab- or internet-based, where the application, position, and design of the FOP have been consistent, simulating mandatory implementation. Voluntary schemes and design inconsistencies may reduce FOP potential to stimulate product reformulation and change consumer behavior [18,34,35,36]. 

### 4.2. Warning Labels Improve Consumer Understanding

The participants in the WL condition demonstrated marked improvements in the understanding of nutrient content compared to the participants in the TLL group. Additionally, the WLs were more effective than the TLLs in reducing the perceived healthfulness of unhealthy products and in identifying the relatively heathier of two or more products. Similar findings have been reported in previously published work. Lima and colleagues found that Brazilian children reduced their healthfulness perceptions of products that displayed WLs and TLLs, while no significant differences between these two formats was found for adults [27]. The WLs have also reduced children’s desire for certain categories of junk food more than the TLLs [24], suggesting better understanding of WLs among children.

### 4.3. Warning Labels Reduce Perceptions of Product Healthfulness

Past studies have found differences between TLLs and WLs for perceived healthfulness but not for their ability to discriminate between products [25]. This may be because of differences in the nutrient thresholds used between studies. The products in this study perhaps displayed a larger number of WLs, leading the participants to further reduce their evaluation of the products’ healthfulness. If this is the case, it raises important implications for the selection of nutrient thresholds for FOP labels and for selecting the categories of products on which to apply them. In the Brazilian context, it would be most appropriate to limit FOP use to ultra-processed products—a category of industrial formulations that are durable, convenient, highly-palatable and contain little or no whole ingredients [37]. The reduction in consumption of these products has been singled out in the Brazilian Dietary Guidelines [38] and by the PAHO [31]. 

### 4.4. Traffic-Light Labels May Hinder Consumer Judgement

While the aggregated results across all products showed small improvements for TLLs in consumer ability to identify excess nutrients, the disaggregated results suggested that, for certain products (soup in this study), the TLL worsened consumer judgment. The reduced performance of the TLL may be explained by differences in the design between labels. The WL was only displayed when the content of critical nutrients exceeded a certain threshold. The TLL was present regardless, with the combination of green, amber, and red nutrients changing across products. The participants made more accurate judgements about excess nutrients in the absence of a label, but contradictory signals of green and amber nutrients on the TLL, in the case of soup, served to override the consumers’ initial judgment. The presence of green and amber nutrients on the same product may have led participants to erroneously perceive the product to be healthier than it was. Similar concerns have been raised in other studies [39,40]. Health agencies may run the risk of confusing consumers by implementing the TLL, potentially reducing its effectiveness in changing consumer behavior.

### 4.5. Warning Labels Shift Consumer Purchase Intentions

In generating a lower mean score of the frequencies and the quantities in which products should be consumed, the WLs appeared to deliver a more consistent message with greater impact on consumer perceptions compared to the TLL. This led to a more conservative consumer choice in this controlled study. Whether these results translate into potentially greater shifts in actual behavior remains to be studied. WLs also elicited significantly stronger substitution effects in this sample—shifting away from intending to purchase the unhealthy products toward the healthier option. A combination of the design, nutrients in ‘green’ on display, and the nutrient cut-off used in the TLL model may have diluted this effect among the TLL participants. Additionally, significantly more people chose not to purchase these products altogether. Here again, the WL outperformed the TLL. The WL influenced changes in choice in another study, where both product abandonment and product substitution were seen, and the observed effects were particularly salient among health-motivated consumers and among product categories where all options displayed at least one WL [41]. 

While similar shifts were reported by Machin and colleagues, no differences between label conditions were found [26]. Labels reduced the number of unhealthful purchases and total food expenditure. As the authors theorized, these shifts may have been due to the fact that the participants were tasked with preparing a healthy meal. When no such health motivation was presented, labels had no effects on purchasing behavior [42]. TLLs and WLs showed no effect in changing purchases of less healthy products or in improving their nutritional composition. In both instances, the product price was included in the simulation, suggesting that the presence of this information along with hedonic factors related to product convenience and taste may dilute the effect of a FOP label, even when it is well understood [14]. Qualitative evidence underscores product price as a priority for consumers, particularly those with financial constraints [43,44]. However, there may be some benefit to labels, irrespective of their design, for consumers concerned about their health. These results warrant replication in other contexts.

### 4.6. Consumer Opinion about Warning Labels Is Favorable

The participants’ general opinion of the WL indicated a higher acceptability of this system, better visibility, and a greater perceived utility than the TLL. The current literature favors the TLL over the WL in terms of participant preference and general opinion on label design. In the qualitative study by Machin and colleagues, mothers stated their approval for the TLL design, their preference for colors, and the ease with which their children could use it [43]. An opinion poll of Brazilian consumers also showed support for the TLL format over the WL (unpublished data). While consumer opinions on FOP labels are important, objective evidence from this and other studies highlight that label appeal may not translate into better objective functioning at time of use.

### 4.7. Study Limitations and Strengths

This study compared the overall label proposal for the WL and the TLL labelling systems, isolating the design effects for just two study outcomes-consumer understanding and label opinions. The survey was a simulation of decision-making and label-related tasks performed in a mandatory labelling scenario, and, while arguably this may be the most efficient method of estimating label effects prior to implementation, it does have its limitations. The study was limited in its ability to replicate the actual shopping experience. Food choices were made in an ideal environment, without the presence of contextual factors or time pressures that may distract the consumer in the real world. Only their intentions to purchase were estimated—consumer purchase behavior in the presence of these labels in the real world may vary. Except for consumer understanding where the design effects of the labels were isolated, purchase intentions may have been influenced by both the differences in design of the FOP labels and their nutrient criteria. Finally, images of the products only displayed the front of the package, and the interaction effects with the nutrition panel or the list of ingredients were not estimated.

The study benefited from a large sample, representative of the Brazilian population on several important demographic factors. The results from the study therefore may be generalizable to a broader population. The randomization of the participants to the label conditions and having the participants serve as their own control ensured that the influence of confounding from known and unknown factors was minimized. Additionally, every effort was made to minimize the bias of the study responses—the survey was entirely anonymous, the participants were blinded to the study aims, and there was no researcher contact at the time of recruitment or data collection. Since WL have not been implemented in Brazil, and this study was conducted before the launch of FOP campaigns, it is also likely that this was the first time that the participants were exposed to the WL. The superior performance of the WL is indicative of the ease of interpretation of this entirely new tool compared to the TLL (different versions of which are voluntarily applied by food manufacturers on a few packaged products in Brazil). The study utilized a variety of products of both single and multiple servings and demonstrated consistent mean effects across a variety of study outcomes.

## 5. Conclusions

In conclusion, the presence of FOP labels on products improved consumer understanding, and perceptions, and influenced purchase intentions. WL was the best FOP label compared to the TLL in helping consumers identify nutrients of concern and in shifting purchase intentions for the product. The WL was more positively rated in terms of design and utility and was also more effective at communicating the need to eat smaller, less frequent quantities of products displaying this label. Potential consumer confusion in the presence of TLL was highlighted. This study provides evidence that a WL would be particularly effective at improving the understanding of nutrient content and the perceptions of product healthfulness, and in shifting purchase decisions in the Brazilian context.

## Author Contributions

Conceptualization, N.K.; Data curation, P.d.M.S., A.P.B.M., and C.G.S.; Formal analysis, N.K.; Funding acquisition, L.A.M., A.P.B.M., and M.T.G.; Investigation, N.K., P.d.M.S., L.A.M., M.T.G., C.F.U.R., and P.C.J.; Methodology, N.K., P.d.M.S., A.P.B.M., C.F.U.R., and P.C.J.; Project administration, L.A.M. and M.T.G.; Resources, A.P.B.M., C.F.U.R., C.G.S., and P.C.J.; Software, C.F.U.R. and C.G.S.; Supervision, P.C.J.; Validation, A.P.B.M. and P.C.J.; Visualization, C.G.S.; Writing—original draft, N.K.; Writing—review & editing, P.d.M.S., L.A.M., A.P.B.M., M.T.G., C.G.S., and P.C.J.

## Figures and Tables

**Table 1:** Survey questions.

Indicator	Survey Question	Response Scale
**Single product task—Participants see images of three products, one at a time**		
Purchase intentions—*Purchase intentions* (*single product*)	(1) Imagine you were looking to buy [type of product]. Would you buy this product or a similar product from a different brand, for yourself or your family?	7-point Likert scale ‘I would certainly not buy’—‘I would definitely buy’
Understanding of nutrient content—*Nutrient content score* (*single product*)	(2) In your opinion, does this product contain certain nutrients in levels higher than recommended for a healthy diet.	Choice of multiple response options: Sugar Sodium Saturated fat Or the response option: None of these nutrients are in excess
Product healthfulness—*Perceived product healthfulness*	(3) Do you think this product is healthy?	7-point Likert scale ‘Not at all healthy’—‘Extremely healthy’
**Product comparison task—Participants see images of two or more products at the same time**		
Purchase intentions—*Purchase intentions* (*comparison task*)	(4) Imagine you were looking to buy [type of product]. Which of these products would you buy for yourself or your family?	Response options for the product pairs: Product A Product B Both products Neither product Response options for the 3-product comparison: Product A (Yes/No) Product B (Yes/No) Product C (Yes/No) All three products (Yes/No) None of these products (Yes/No)
Understanding of nutrient content—*Nutrient content score* (*comparison task*)	(5) Which of these products has a larger quantity of the following nutrients: sugar, sodium, saturated fat.	Response options for the product pairs: Product A has more of this nutrient Product B has more of this nutrient Both products have high levels of this nutrient Both products have low levels of this nutrient Response options for the 3-product comparison: Product A Product B Product C These three products have high levels of this nutrient These three products have low levels of this nutrient
Product healthfulness—*Product healthfulness score*	(6) Please choose the product you think is relatively healthy.	Response options for the product pairs: Product A is healthier Product B is healthier Response options for the 3-product comparison: Product A Product B Product C
**Label only task—Participants see the image of the label only**		
Label understanding	(7) In your opinion, how frequently should a product with this label be consumed?	7-point Likert scale Never—Always
Label understanding	(8) In your opinion, in what quantities should a product with this label be consumed?	7-point Likert scale In small quantities—In large quantities
Purchase intentions	(9) What would you do if you saw this label on a product that you usually buy?	7-point Likert scale I would not buy it—I would continue buying it
Label opinions	(10) The label on the product draws my attention. (11) The label on the product is not visible. (12) I think this label is easy to understand.	7-point Likert scale Totally disagree—Totally agree
	(13) This label will help me quickly decide what products to buy.	
	(14) I think that this label will not help me identify more healthy food.	
	(15) This label will help me decide whether or not to buy a product.	
	(16) I consider the information on this label credible and true.	
	(17) This label will not change my decision about what products to buy.	

**Table 2:** Demographics.

Indicators	Total Sample *n* = 1607	Traffic-Light Label *n* = 804	Warning Label *n* = 803	Comparing between Label Conditions *p*-Value
Age, mean years (SD)	39.27 (12.94)	39.24 (13.04)	39.29 (12.86)	0.936
Weight, mean kgs (SD)	74.54 (26.64)	74.93 (32.89)	74.15 (18.37)	0.557
Sex, %				
Female	52.46	52.24	52.68	
Male	47.54	47.76	47.32	0.860
Age group, %				
18–34 years	40.20	40.17	40.22	
35–54 years	44.99	45.27	44.71	0.951
>55 years	14.81	14.55	15.07	
Education, %				
Primary or less	13.19	13.43	12.95	
Secondary	68.89	67.66	70.11	0.525
Tertiary	17.92	18.91	16.94	
SES, %				
Low	14.87	15.42	14.32	
Medium	47.92	46.14	49.69	0.363
High	37.21	38.43	35.99	
Geographic region, %				
North	7.59	6.84	8.34	
North-east	17.80	17.29	18.31	
South	17.42	17.41	17.43	0.747
South east	47.17	48.38	45.95	
Mid-west	10.02	10.07	9.96	
With CVD diagnosis, %	18.67	17.07	20.30	0.094
With diabetes diagnosis, %	22.03	20.65	23.41	0.181
Currently dieting, %	31.11	27.86	34.37	0.005

SD: Standard deviation; SES: Socio-economic status; CVD: Cardio-vascular disease.

**Table 3:** Performance on study outcomes between control and label conditions.

Outcome	Control Condition *n* = 1607	Label Condition *n* = 1607	*t*-Test Statistic
	**Mean (SD)**		
**Nutrient content score (single product)** **(0–100)**	51.08 (38.82)	68.73 (38.86)	−12.92 *
**Nutrient content score (comparison task)** **(0–100)**	39.40 (26.45)	53.40 (39.54)	−17.21 *
**Product healthfulness**			
Perceived product healthfulness 1 ‘Not at all healthy’–7 ‘Extremely healthy’	3.15 (1.51)	2.52 (1.39)	19.99 *
Product healthfulness score, (0–100)	64.54 (17.38)	71.21 (15.74)	−15.55 *
**Purchase intentions (single product)** 1 ‘I will certainly not buy’–7 ‘I will certainly buy’	4.66 (2.63)	3.28 (1.70)	35.31 *

* *p* < 0.001.

**Table 4:** Performance on study outcomes between traffic-light label and warning label conditions.

Outcome	Control Condition (T1)			Label Condition (T2)			Difference between WL and TLL in Change from T1 to T2
	**Traffic-Light Label**	**Warning Label**	**Test Statistic *p*-Value**	**Traffic-Light Label**	**Warning Label**	**Test Statistic *p*-Value**	**Test Statistic *p*-Value**
	***n* = 804**	***n* = 803**		***n* = 804**	***n* = 803**		
	**Mean (SD)**			**Mean (SD)**			
**Label understanding**							
Frequency of consumption 1 ‘Never’–7 ‘Always’	-	-	-	3.50 (1.43)	2.13 (1.43)	F 366.22 <0.001	-
Quantities of consumption 1 ‘In small quantities’–7 ‘In large quantities’	-	-	-	2.74 (1.57)	1.59 (1.14)	F 280.94 <0.001	-
**Perceived product healthfulness, single product**							
1 ‘Not at all healthy’–7 ‘Extremely healthy’	3.20 (1.53)	3.09 (1.48)	F 2.13 0.144	3.02 (1.46)	2.02 (1.11)	F 240.19 <0.001	F 231.84 <0.001
**Purchase intentions**							
Purchase of a frequently bought product 1 ‘I would not buy it’ 7–‘I would continue buying it’	-	-	-	3.59 (1.87)	2.04 (1.36)	F 356.46 <0.001	-
Purchase intentions, single product 1 ‘I will certainly not buy’–7 ‘I will certainly buy	4.67 (1.64)	4.65 (1.63)	F 0.06 0.799	3.94 (1.68)	2.61 (1.45)	F 288.43 <0.001	F 338.93 <0.001
**Positive label opinion**							
1 ‘Disagree’–7 ‘Agree’	-	-	-	4.53 (0.89)	5.09 (0.87)	F 165.26 <0.001	-
